# The Urban Deployment Model: A Toolset for the Simulation and Performance Characterization of Radiation Detector Deployments in Urban Environments

**DOI:** 10.3390/s24154987

**Published:** 2024-08-01

**Authors:** Nicolas Abgrall, Yassid Ayyad, Chun Ho Chow, Reynold Cooper, Daniel Hellfeld, Emil Rofors

**Affiliations:** Lawrence Berkeley National Laboratory, 1 Cyclotron Road, Berkeley, CA 94720, USArjcooper@lbl.gov (R.C.); erofors@lbl.gov (E.R.)

**Keywords:** detector array, network, urban environment, national security, nuclear threat

## Abstract

Static and mobile radiation detectors can be deployed in urban environments for a range of nuclear security applications, including radiological source search-and-tracking scenarios. Modeling detector performance for such applications is challenging, as it does not depend solely on the detector capabilities themselves. Many factors must be taken into consideration, including specific source and background signatures, the topology and constraints of the deployment environment, the presence of nuisance sources, and whether detectors are mobile or static. When considering the simultaneous deployment of multiple, heterogeneous detectors, assessment of the system-wide performance requires the simulation of the individual detectors, and a system-level analysis of the detection performance. In radiological source search-and-tracking scenarios, performance is mostly dominated by the probability of encounter, which depends on the specifics of a given deployment, e.g., static vs. mobile detectors or a combination of both modalities, the number of detectors deployed, the dynamic vs. static setting of false alarm rates, and individual vs. networked operation. The Urban Deployment Model (UDM) toolset was specifically developed to cover the gap in the available generic frameworks for the simulation of radiation detector deployments at city scales. UDM provides a unified and modular framework to support the simulation and performance characterization of heterogeneous detector deployments in urban environments. This paper presents the key components along the UDM workflow.

## 1. Introduction

The effective use of detection assets and personnel is critical for ensuring a nation’s capability to deter, detect, and report unauthorized attempts to either import, possess, store, develop or transport radioactive/nuclear material. Recent work has investigated distributed and networked sensors as a feasible capability to effectively detect, identify, and track sources through large city-scale areas [1,2,3,4]. Improving the fidelity with which radiation detector deployments are simulated allows for improved mission planning, and more informed decision making. It can also help to identify gaps in current capabilities, and inform the development of next-generation technologies. In the urban search scenario, in which multiple technologies are brought to bear in highly complex environments, high-fidelity modeling is especially needed to capture the myriad factors that influence the performance of any individual or set of detectors.

Proper modeling should first provide representations of specific cities at large scale, with a level of detail high enough to accurately simulate the transport of detectors and sources through the environment, as well as the direct impact the environment has on the detection performance (e.g., occlusion from buildings, and multiple traffic lanes). Second, physics-based modeling should be used to describe the detector response to source signatures, and a potentially complex spatially varying radiological background for multiple detector types.

Proper modeling of the environment and detector responses are key to produce synthetic data streams representative of the simulated detector systems. However, the detection, isotope identification, and localization performance of a given system also highly depend on the choice of the corresponding algorithms. The latter should be chosen/approximated appropriately based on the simulated detector types for relevant comparisons.

## 2. The Urban Deployment Model

**Figure 1:**
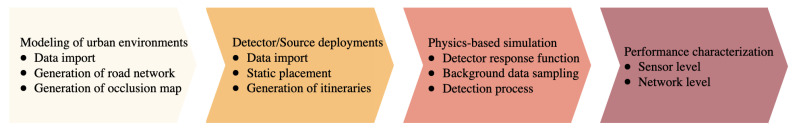
The Urban Deployment Model (UDM) workflow: a series of steps guides the simulation setup through all major components.

A wide range of fidelity is possible for the simulation of radiation detector deployments in urban environments. Some aspects of the simulation may require only simple approximations of the physical processes involved, while others need a high level of detail to reach any reasonable accuracy in performance estimation. To cover all the components of the simulation of these deployments, the Urban Deployment Model (UDM) toolset was developed with a workflow (Figure 1) that builds upon each key component. In the following sections, we provide an extensive description of each of these, from minimal approximation to ideal case, and discuss possible software implementations. The components are grouped under the following four stages of the simulation process:

- Modeling of urban environments;
- Static and mobile deployments;
- Physics-based simulation of the detection process;
- Performance characterization.

## 3. Modeling of Urban Environments

Complex urban environments significantly impact the detection performance of deployed systems in any source search-and-tracking scenarios. The local environment primarily defines how sources and detectors move relative to each other, whether a source is occluded from any detectors at any time, and the nature of the radiological background. Deployment simulations solely based on analytical formulations of detection probabilities (e.g., as a function of the covered area, system density, and detection range) cannot capture any of these aspects. It is thus necessary to consider agent-based simulations, with specifically modeled environments rather than generic urban models (e.g., couple of city blocks). Some open-source data that capture many of the static and dynamic aspects of urban environments can be leveraged for modeling. Of particular interest are the data queried from the OpenStreetMap (OSM) [5] and GoogleMaps [6] servers.

Among all factors that can impact the detection efficiency of a set of deployed detectors in urban environments, the following were found to be the most relevant.

### 3.1. Road Network

The OSM data set provides a representation of the road network in the form of geospatial nodes linked together in such a way as to approximate the real-world roadways. When limited to motor vehicles, the data comprise different roadway levels, from service and residential roads, primary and secondary axes, to freeways. Parsing these roadways provides a first approximation for an agent-based type of simulation, where detectors and sources can be transported on the road network.

In UDM, a greater level of accuracy can be achieved by parsing additional attributes of the roadways, such as direction, and number of lanes. The latter, in particular, is used to reconstruct the proper relative position of different lanes along the roadways, which significantly impacts the distance of approach between sources and detectors.

Concerning possible implementations, several libraries (e.g., [7,8]) are available to parse OSM data. Due to the format of the roadway data and the nature of an agent-based simulation, a directed graph is a very suitable structure for the representation of the road network. Several graph libraries are available, sometimes directly integrated with OSM parsing libraries [7,9,10]. UDM leverages some of these libraries to build a directed graph of the road network over the region of interest for any combination of roadway types. The graph accounts for multiple lanes (distinct edges of the graph), and properties such as distance and local speed limits are stored at the edge and node levels as inputs to graph traversal and optimization algorithms.

### 3.2. Occlusion

In complex urban environments, a source can be temporarily occluded from the line of sight of a detector by both static and dynamic components. Among others, static components include buildings, road network structures such as tunnels and bridges, urban equipment, and parked vehicles. The main dynamic component consists of vehicles passing by the source or detector. While an extensive simulation might try to model all of these components, a minimal implementation should account for static components like buildings, tunnels, and bridges, as high-density construction materials will attenuate the radiation signal most significantly. The OSM data provide some information that can be used to model occlusion from these components. In particular, building footprints and heights can be retrieved, while a layer attribute encodes the layout of roadways with respect to ground level (i.e., above/under ground level).

A high-detail physics-based simulation of radiation transport would account for attenuation due to different material types and thicknesses, and for the down-scatter contribution from surrounding materials in the environment. While the latter can somewhat be approximated by including ground and/or surrounding materials in the simulation of detector response functions, the former requires additional building material information that is not readily available. A possible option is to provide a typical material composition (e.g., concrete, iron, and glass) expected in urban environments, and use the corresponding attenuation coefficients to provide an effective attenuation for the thickness of material traversed. However, because dynamic occlusion times are relatively short compared to the total typical time of a source encounter, a simple binary occlusion model is a reasonable first step to start with. In such a model, radiation that would reach a detector from a source is simply discarded if any occluding element lies on the line of sight between the two.

The default occlusion model in UDM is such a binary model. The implementation is based on a balanced-tree data structure that stores bounding boxes for the polygons corresponding to the building footprints in OSM data. Such a data structure can efficiently be queried for intersection and overlap between geometrical primitives and is used to suppress signal upon occlusion. The UDM graph representation also stores the road network layer information mentioned above at the node level, which is used to suppress the signal from occlusion due to the source and detector being on different levels (e.g., ground level and tunnel or bridge). As a first approximation, sources and detectors on the same level are considered to be located at the same standoff from the ground, distances and occlusion being effectively estimated on a plane. Should they require different standoffs, the occlusion model will account for building heights as well estimating distances and occlusion in 3D Cartesian coordinates.

### 3.3. Traffic Dynamics

Considering that threat sources would be transported by vehicle to potential target sites in urban environments, local traffic dynamics plays an important role in the simulated spatial and temporal encounter distributions between sources and both dynamically and statically deployed detectors. Indeed, depending on its mode of operation, simulating a set of statically deployed detectors might as well require realistic traffic data input. Studies found in the literature (e.g., [11,12]) concentrate on dense arrays of independent sensors over small-scale areas, where realistic traffic dynamics does not impact performance at the sensor level. However, some studies have exposed the concept of using the network capability of statically distributed sensors (e.g., [13]) for sparse arrays deployed over city scales. The detailed performance study of this network operating mode requires precise estimations of the time of travel between relevant detector locations to correlate signals and possibly track threats through the environment. It is thus important that simulated itineraries for threat sources and mobile detectors through the modeled environment account for realistic traffic dynamics. Several data sources are available to simulate local traffic. As a first approximation, average or local speed limits such as those provided in OSM data can be used, but those do not capture the variations observed in real traffic flow and density. Using itinerary and travel time estimates from services such as GoogleMaps offers much better fidelity. Common transport platforms, like Uber or Lyft, have also made some of their pick-up and drop-off locations public (see [14] for an example use case). Finally, several cities make their public transportation data available, for example, as real-time GPS locations of their bus fleets. UDM can import any of the previous sources to generate corresponding itineraries on its multi-lane graph representation of the road network. In the particular case of GoogleMaps data, UDM provides a wrapper around the native API [15] to query travel times and shortest-time itineraries. Query results from the GoogleMaps servers are by default based on historical data, which are sufficient for most cases, but one notes that commercial accounts are given access to real-time traffic data.

## 4. Modeling of Mobile and Static Detector Deployments

### 4.1. Mobile Detector Deployments

Two broad categories of deployments are considered in UDM: *itinerary*-based and *search* deployments. The itinerary-based deployments include all cases where detectors follow a route from point A to point B, possibly including a set of way points. Whether the start and end points, or series of way points are randomized or obtained from any of the data sources mentioned in Section 3.3, UDM generates corresponding itineraries on its graph representation by leveraging libraries implementing graph traversal algorithms, such as Dijkstra’s [16] or the A* (A-star) [17] shortest-path algorithms. Based on estimated travel times and/or local speed data, positions along the itineraries are spatially sampled according to the specified acquisition rate of the simulated detectors. Thus, real traffic patterns can be captured from data to increase the fidelity of the simulation. One notes that the same tools are used to simulate the itinerary of threat sources traveling through the environment.

The search deployments cover cases where one or several detectors are deployed over a specific region to possibly detect or eliminate the presence of a static source as fast as possible. UDM enables such deployments by implementing them as graph-based optimizations, effectively solving the postman tour/route inspection problem [18]. The construction of a local Euler graph makes it possible to compute the visitation pattern of the graph vertices that minimizes the traversal rate of each edge of the graph, thus providing a time optimization of the search path that a detector needs to follow to clear the area.

### 4.2. Static Detector Deployments

As the probability of source encounters over time mostly drives the detection performance of a given deployment, it is important to optimize the placement of static sensors to maximize encounters. For scenarios where a region of interest is to be monitored, the ideal optimum is simply to provide uniform coverage. However, depending upon the size of the region of interest, the number of detectors required to do so quickly becomes prohibitive. Several factors can be considered to reduce that number; for example, the placement can be optimized relative to traffic density patterns or to how often certain locations are passed by.

**Figure 2:**
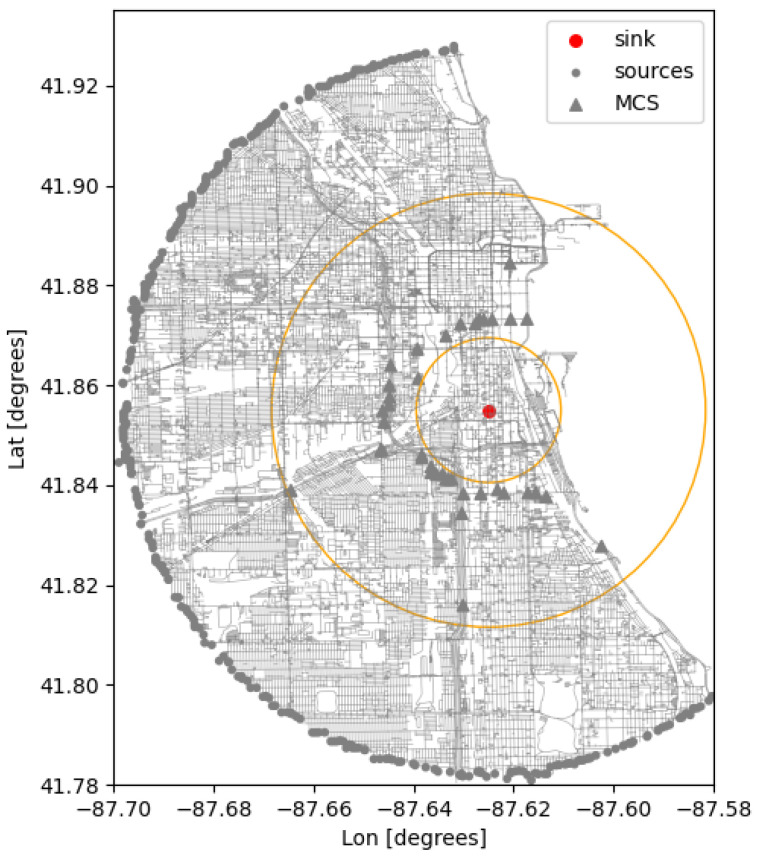
Minimum cut set (MCS) calculation: a cut ring (orange circles) is centered around the sink vertex with inner and outer radii of 1 and 3 miles, respectively. Sources are taken to be all possible inbound vertices within the region of interest. The MCS consists of 39 edges, some identifying clear choke points along major arteries.

In a *target protection scenario*, static detectors are deployed around a target location to monitor incoming traffic up to a minimal distance of approach, the goal being to prevent any threat from reaching that minimum distance to the target undetected. Graph theory provides an elegant solution to the problem with the *max-flow min-cut* theorem, applicable to graphs called *flow networks* [19]. The directed edges of such graphs are assigned flow *capacities*, which effectively determine how much flow can circulate from a *source* to a *sink* vertex. It is possible to construct one or several sets of edges that cut the graph in such a way that the source and sink vertices end up in disconnected partitions of the graph, i.e., no flow can circulate anymore from source to sink. These sets are called *cut sets*, and the value of a cut set is defined as the sum of the capacities of its edges. The max-flow min-cut theorem states that the maximum flow that can circulate from source to sink is equal to the minimum value taken over all the possible cut sets. The set of edges constituting the cut set with the minimum value is referred to as the *minimum cut set* (MCS). The computation of an MCS on the graph representation of the road network can provide deployment optimization by identifying choke points on the network where sensors can be deployed for an increased encounter probability. The calculation of the MCS locations in UDM is based on the implementation of the *Boykov–Kolmogorov max-flow algorithm* from the Boost Graph Library [10]. An illustration of such a computation is given in Figure 2. One notes that the distance-based computation currently implemented does not capture traffic dynamics. By construction, a time-based computation using time of travel estimates would account for such effects. The placement optimization could then be based on a typical threat response time with respect to the target location.

## 5. Physics-Based Simulation of the Detection Process

### 5.1. Simulation of Detector Responses

As most detector systems show an angular dependence in their detection efficiency, it is important to model their response functions to different isotopes over the appropriate angular range. Physics-based simulations of gamma radiation–detector interactions, such as those performed using Geant4 [20], can provide accurate energy responses to different detector geometries, sources, and relative placements of the source and detector. The accuracy of such models does, however, come at a computational cost that is not suitable for the estimation of the detector response at each timestamp of a deployment simulation. A common workaround is to pre-generate the detector response functions over a discrete two-dimensional grid in varying energies and angles of incident radiation, and to rely on a much more computationally efficient interpolation during simulation. Thus, two main factors drive the accuracy of physics-based detector response functions for deployment simulations: the extent of the modeling for the pre-generated responses, and the corresponding interpolation and sampling methods used at run time.

The modeling covers the following components:

- **Geometry:** To a first approximation, only the geometry corresponding to the sensitive volume of the detector can be modeled. However, the more detailed the geometry, the more realistic the generated response. Thus, any additional detector material around the sensitive volume should be modeled to properly account for attenuation. This consideration extends to the medium the incident radiation propagates in, air being more accurate than vacuum.
  The surrounding geometry, such as ground plane and buildings, is necessary to recreate non-detector effects in the observed response, due to the incident radiation being down-scattered in these materials, and redirected toward the detector. However, such considerations require a significant increase in computation resources, which is commonly prohibitive. A compromise between the inclusion of some scattering geometry and a reasonable computation time may be found.
- **Coverage:** In urban scenarios, the encounter configurations between sources and detectors are often such that the distance between them is large enough to consider a far-field approximation. In this case, incident radiation can be simulated as a non-divergent beam (i.e., parallel rays) with a transverse size large enough to cover the projected surface of the detector volume over all considered angles.
  The angular coverage in polar and azimuthal angles should ideally correspond to the full 4π solid angle around the detector. However, some considerations about the detector symmetry might allow to limit the coverage to half or a quadrant of that. Several methods can be used to distribute points on a sphere, and thus define the standoff positions populating the angular grid. In particular, the HEALPix [21] representation provides uniform sampling on the sphere with increasing density levels (e.g., simulating 192 beams being a reasonable choice for smoother interpolation between angular positions).
  The energy coverage should be large enough to include primary gamma-ray lines of common isotopes and sources of interest, as well as accounting for the detector dynamic range. Simulating beams with energies over the 50 to 3000 keV range in steps of 50 keV is a good baseline.
  The simulated statistics obviously contributes to the accuracy of the generated response when normalized per emitted particle at each angle–energy point of the grid. The number of incident rays per point should be in the 1E6 range to achieve reasonable accuracy.
- **Resolution:** The simulated detector responses need to be blurred to account for detector resolution. An energy-dependent blurring function, such as a Gaussian kernel defined as $FWHM\left(E\right)=A^{2}+B^{2}E+C^{2}E^{2}$, can be applied to the generated spectra for that purpose, the *A*, *B*, and *C* detector-dependent parameters being estimated from a fit to experimental data.

**Figure 3:**
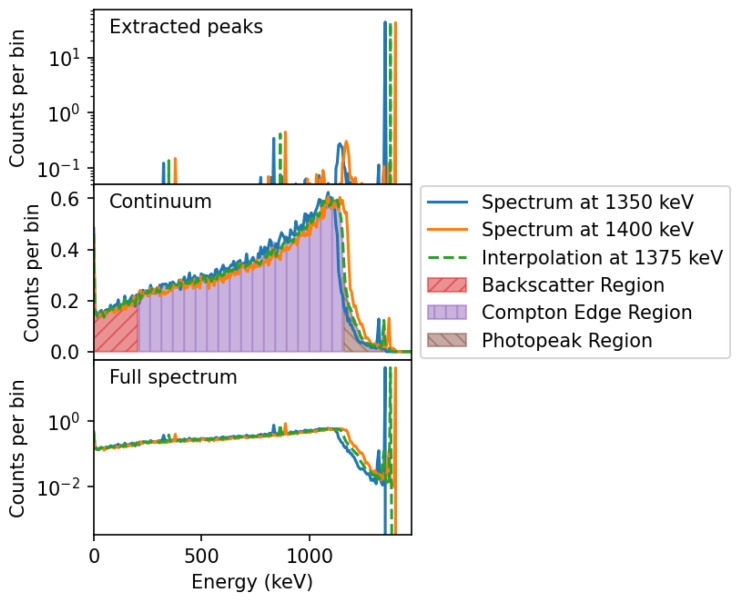
Two simulated energy depositions in an NaI crystal from gamma rays of 1350 keV and 1400 keV, and the interpolated spectrum at 1375 keV. The top plot shows the photopeaks, and the middle plot shows the continuum spectra, which are interpolated separately. The bottom plot shows the full spectra and resulting combined interpolated spectrum.

The energy interpolation requires more care to produce good results, and it was found that treating different regions of the spectra separately is more appropriate as shown in Figure 3. The first step consists of removing photopeaks and potential positron–electron annihilation peaks from the spectra, and interpolating them to the target energy as shown in the top plot. The remaining continuum part of the spectra is scaled to the requested energy individually in three regions: below the back-scattering energy, above the Compton edge, and in between. The scaled continuum spectra are combined, weighted by how close to the target energy the original spectrum is, and the interpolated photopeaks and untouched annihilation peaks are added back into the resulting spectrum, which is shown in the bottom plot of Figure 3.

Using this methodology, the detector response matrix can be used to pre-generate templates for any source of interest. Indeed, the energies, branching ratios, and relative intensities of the primary gamma-ray lines of common isotopes are available in the literature, or can also conveniently be queried from databases such as the National Nuclear Data Center (NNDC) [22]. This information can readily be used to combine interpolated spectra at the proper energies over the full angular coverage. Thus, at run time, the observed response for a given source at a particular position relative to the detector can efficiently be interpolated, Poisson sampled, and scaled by the source-to-detector distance ($1/r^{2}$ law), the source activity (counts/s), and the duration of the time step. The detector responses generated following this approach can only be used to provide bare source templates, in the sense that the geometry of the source itself is not accounted for. In particular, any shielding material would significantly affect the expected spectral features of the source. However, computation resources permitting, templates for specific shielding material and geometry could certainly be generated if such information were available.

### 5.2. Background Sampling

Simulating the radiological background that a mobile or static detector would observe in an urban environment requires to simulate the deployment environment at a level of detail high enough to account for all the complexity arising from the spatial- and time-varying nature of a realistic background. Even though the simulated environment will certainly differ, and thus be subject to different spatial variations and correlations, sampling the background from real data acquired with the detector being simulated, or a similar system, is a simpler approach. Background data might be available from different acquisition modalities:

- **Time series:** Counts or spectra for systems with spectroscopic capabilities. In this case, counts/spectra can be sampled along their time series, thereby conserving time correlated features between samples. Another approach is to Poisson sample counts from the mean gross count rate or average spectra.
- **Distributions:** A gross count rate distribution, or the experimentally determined mean and variance of such a distribution, can be used to sample gross counts. A fit to the data with a Poisson distribution provides good adequacy for smaller volume detectors, while a negative binomial distribution usually fits better for larger volume detectors. Indeed, for larger volumes, the addition of a second variance term accounting for deviations from the Poisson distribution due to clutter in the environment captures better the fact that a larger volume detector with higher count statistics effectively samples many Poisson distributions simultaneously.

In an urban environment, not all background sources are expected to be isotopes naturally occurring in the environment and construction materials (e.g., ^40^K, NatU, ^232^Th, ^226^Ra). The background may also include isotopes that are commonly used for medical (e.g., ^99*m*^Tc for PET scans) and industrial (e.g., ^137^Cs in density gauges) applications, and may be observed in the detectors during sporadic pass-by encounters. These types of sources are usually referred to as *nuisance* sources. Physics-based detector responses can be generated for these nuisance sources and used to inject signals during simulation. However, how to perform source injection realistically is not obvious, as there exist very little data about the patterns of life these nuisance sources typically follow.

### 5.3. The Detection Process

**Figure 4:**
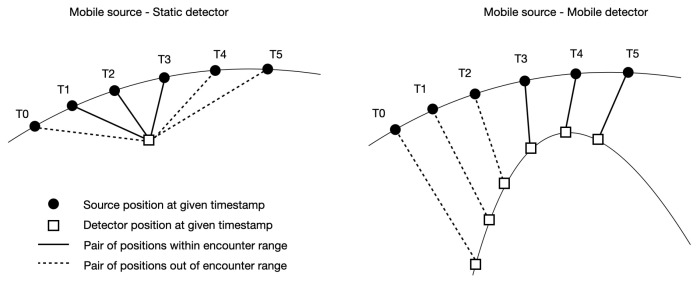
(**left**) Time-consecutive positions of a source and static detector during simulation. (**right**) Same for a dynamic detector. The pairs of positions linked with a solid line constitute an encounter.

In an agent-based simulation, the positions of the deployed systems are calculated at each timestamp based on their dynamics and the constraints from the local environment. For any given pair of sources and detectors, their time-aligned positions may end up being in proximity within a given distance for any time-consecutive series of steps (see Figure 4). Such a series is referred to as an *encounter*. The encounter distance is typically defined such that it is much larger than the maximum detection range of the simulated detector systems.

The detection process consists in determining whether a detection occurred or not during an encounter. Depending on the information available about a given system and/or the desired level of accuracy for the simulation of the encounter dynamics, different versions of the detection process can be implemented:

- **Range-based detection:** This is a simple binary implementation, where a detection is recorded if for at least one of the pairs of time-consecutive positions of the encounter, the corresponding distance is within the detector *range*.
  The detector range refers to the standoff distance (or distance of closest-approach) at which the probability of detection has a certain value. Vendors commonly quote detector ranges corresponding to 50 to 90% detection probabilities. This value intrinsically depends on several parameters such as the activity of the source, the relative speed between the source and detector, and the false alarm rate at which the detector is operated. When simulating the deployment of different detector systems and using a range-based detection process, it is useful to estimate their detection range within a common measure. Indeed, some systems may have spectroscopic capabilities, while some others may rely only on counts within a given energy window. Furthermore, as different detectors will have different responses to background, direct comparisons of their detection performance should be conducted at a common false alarm rate. With these two constraints in mind, estimating detection ranges with a simple algorithm performing on detector counts within a given energy window, is a good compromise between including detectors with and without spectroscopic capabilities, and possibly penalizing those with such capabilities by underestimating their detection range. The *k-sigma* algorithm is one such algorithm that, for a given false alarm rate, computes the detection threshold in terms of the number *k* of standard deviations (σ) above the mean background rate (μ). For a given pair of source and detector positions, detection occurs if the observed count rate in a determined energy window *N* is such that (N−μ)/σ>k.
  If not experimentally determined, detection ranges can be estimated from *pass-by* simulations: for a nominal source activity, detector speed, and false alarm rate, the detector position is calculated at consecutive timestamps (e.g., 1 s integration time) along a linear trajectory at a given standoff distance from the static source. Both the background and physics-based detector response to the source must be sampled at each timestamp. A detection is recorded for a given standoff distance if, for any position along the trajectory, the total number of counts above the mean background is above the k-sigma threshold. By repeating this simulation a large number of times for a range of standoff distances, one constructs the curve representing the average probability of detection as a function of distance, from which one can estimate the detection range at a given probability.
- **Signal-based detection:** In this case, both background and detector response are sampled and combined at each timestamp. This data stream is passed to the appropriate detection algorithm for the simulated system to determine whether a detection occurred or not during the encounter. Contrary to the simpler range-based detection method, this approach is not bound to a given source activity or relative speed, and will not underestimate the detection performance by using a non-optimal detection algorithm for the considered system.

### 5.4. Experimental Validation

**Figure 5:**
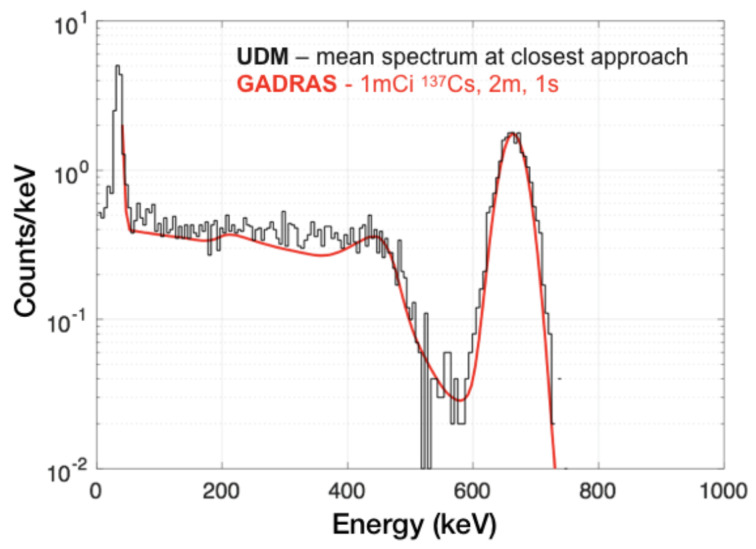
Comparison between the average 1 s spectrum generated from 100 UDM simulations of a 1 cubic inch CsI detector observing a 1mCi ^137^Cs source from 2 m (black), and an independent GADRAS simulation of the expected detector response in the same configuration (red).

Experimental validation of the simulated performance of large-scale deployments can only be very limited due to the lack of proper experimental data. Such validation would indeed require controlled pass-by/drive-by runs with sources in urban environments with deployed static and/or mobile sensors. However, the overall source injection chain of UDM was benchmarked against both reference simulation programs and experimental data. Those benchmarks validate in particular the UDM detector response generation and interpolation. As an example of benchmark against reference simulation programs, Figure 5 shows a comparison between a GADRAS [23] generated source spectrum for a 1 cubic inch CsI detector (1 s dwell time) and a 1mCi ^137^Cs source located at 2 m and 0 degree angle, and the mean spectrum obtained from 100 pass-by injections in UDM. Each pass-by consists of a series of sampled positions along a straight line with the source located 2 m away from the point of closest approach. The positions are sampled according to the detector speed and dwell time. The source spectrum is obtained at each sampled position after interpolation from the Geant4 response functions, scaling for the dwell time and source activity, and Poisson sampling. The mean spectrum shown in Figure 5 is averaged at the point of closest approach, effectively corresponding to a distance of 2 m and angle of 0 degrees. Excellent agreement is obtained with the GADRAS generated spectrum.

**Figure 6:**
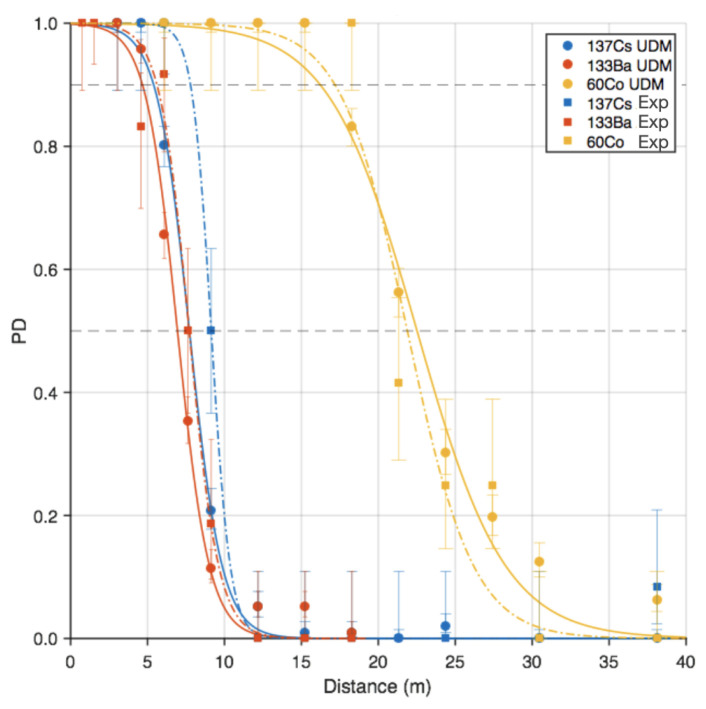
Experimental and simulated range curves for a CZT device with detection threshold set for an estimated probability of false alarm of $2\times 10^{-5}$. Simulated/experimental data points are represented by solid circles/squares and the fits to the simulated/experimental data points by solid/dashed lines. The error bars on the data points correspond to 50% binomial confidence intervals associated with the calculated values for the probability of detection (PD).

An example of benchmark against experimental data is shown in Figure 6, comparing measured and simulated range curves for a CZT (CdZnTe) device. The statistical uncertainties are relatively small in the simulated data, as 100 trials were conducted at each distance. The uncertainties associated with the experimental data are significantly larger given the limited number of trials. Discrepancy between the measured and simulated detection range values at a probability of detection (PD) of 0.5 were estimated to 10.4% for ^133^Ba, 15.4% for ^137^Cs, and 2.7% for ^60^Co. Those are likely attributable to uncertainty in the experimental distance of the closest approach and the inability of the simulations to fully reproduce the specific scattering environment of the experimental measurements. However, Figure 6 shows an overall very good agreement between the empirical data and the UDM simulations.

### 5.5. Scalability

The UDM toolset aims to simulate large-scale deployments in urban environments. At those scales, the local varying traffic and occlusion conditions are not as important as their cumulative effect along itineraries through the environment. UDM can capture global varying traffic conditions by generating routes for mobile sensors and sources using travel time estimates and shortest-time itineraries queried from GoogleMaps. By sampling the background from real urban data, UDM also captures some of the spatial and time variability in background due to obstructions. However, the impact of such effects on the source detection process is not accounted for by default. Some of them could be implemented to some extent, for example, by randomizing source stops at traffic lights/intersections (data from OpenStreetMap) and by introducing random obstructions along the line of sight between sources and detectors. Although nothing in the UDM workflow prevents building a small-scale urban environment, the uncertainties coming from the lack of, or limited implementation of such local dynamic effects, will become larger the smaller the domain becomes.

## 6. Performance Metrics

Performance estimations for a given deployment depend on the level of detail applied to the simulation of the urban environment (road network, occlusion, and traffic dynamics), and the physics-based detection process. As described in the previous sections, there exists a whole range of detail, and the highest level should always be used to estimate the performance as accurately as possible. A metric commonly used to assess the performance of a deployed array of detectors is the *probability of detection* for a specific source isotope and activity. For a given number of routes followed by the source of interest passing through the array, the probability of detection is simply defined as the ratio of the number of routes for which the source is detected to the total number of routes. The cost of detector units and the false alarm rate that can be tolerated over the array being major constraints, it is common to study the probability of detection as a function of the number of units in the array for different deployment strategies (e.g., static and/or mobile units, and different detector types).

In this section, we present a simple deployment simulation to illustrate some of the concepts described in this paper.

**Figure 7:**
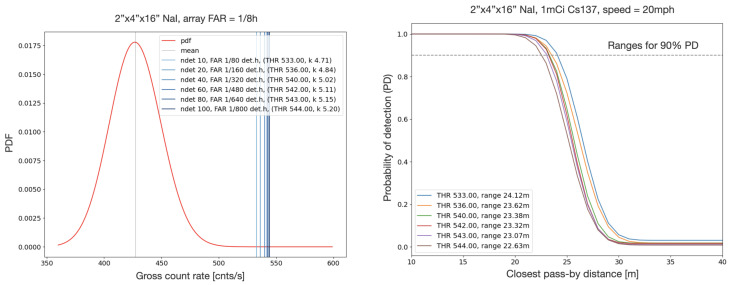
(**left**) NaI detector thresholds. (**right**) Range curves. Thresholds and range curves are estimated for different values of the false alarm rate. Ranges are quoted for a 90% probability of detection.

**Figure 8:**
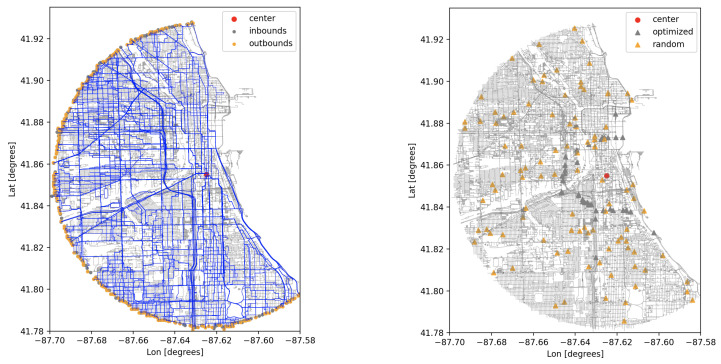
(**left**) Simulated routes over the covered region. (**right**) Static detector placements.

- **Detector type:** 2”x4”x16” NaI(Tl) crystals.
- **Detector response function:** Geant4 simulation, with energy and azimuthal angle interpolations.
- **Source:** ^137^Cs, 1mCi.
- **Detection process:** range-based detection. The mean and variance of the background gross count rate distribution are estimated from experimental spectroscopic data, and used to compute thresholds for different false alarm rates based on the corresponding negative binomial distribution (see Figure 7, left). Pass-by simulations are performed to estimate the detection range for a 90% detection probability as a function of the false alarm rate (see Figure 7, right).
- **Urban environment:**
  - Road network: region covers a 5mi radius around a central location, and includes all traffic ways for motor vehicles. Both single and multiple lane approximations are used.
  - Occlusion: no occlusion model.
  - Traffic dynamics: using an average speed of 20 mph. A total of 200 routes are generated over the covered region with a shortest-path algorithm. Routes start and end at randomly selected inbounds and outbounds, and pass through the central location, after and before reaching two randomly selected pass-by points (see Figure 8, left).
- **Deployment type:** Static units. A first set of 100 units is randomly deployed over the covered area. A second deployment is optimized using a minimum cut set calculation relative to the central location, and consists in 39 units (see Figure 8, right).

**Figure 9:**
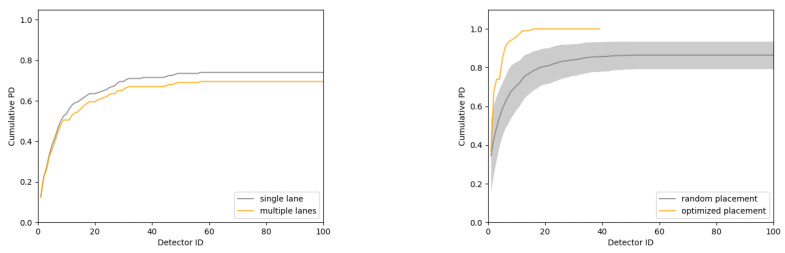
(**left**) Cumulative probability of detection for single and multiple lane approximations. (**right**) Cumulative probability of detection for random and optimized static placements. The error band for the random placement shows the standard deviation over 100 trials.

We first assume no information on threat patterns is available. In this case, a uniform coverage of the region of interest may be a good strategy, depending on the total number of units required to do so. We approximate this scenario with the deployment of 100 static units at randomly selected locations. To demonstrate the impact that the level of simulation of the road network can have, we estimate the cumulative probability of detection of the array using both single and multiple lane approximations. The cumulative probability is obtained by ordering the units in the array in a decreasing number of detections. As can be seen in Figure 9, left, for this range-based detection scenario, the simple single lane approximation can overestimate the probability of detection by 5%. This number will vary depending on the scenario parameters such as the source strength and the type of detection process being simulated (i.e., range- vs. signal-based detection).

In a second scenario, we consider the fact that routes are constrained to pass by the central location of the covered region. In this case, we deploy the minimum number of units (39) required to constitute a cut set within 1 to 3 miles from the central location. Figure 9, right, shows a performance comparison with an ensemble of random placements. This illustrates the importance of optimizing placement for a significantly increased probability of detection, with a decreased number of units, and a lower deployment cost.

## 7. Conclusions

Various applications in nuclear non-proliferation, homeland security, and basic science require the use of statically and dynamically deployed radiation detectors to monitor background radiation, and detect and identify anomalous signatures. In the context of large city-scale sparse deployments, it is being understood that domain awareness (from contextual sensors) in conjunction with networked operation is required to provide enhanced detection performance. In order to study/optimize the operation and performance of such deployments, a simulation framework should support high-fidelity physics-based simulations. The Urban Deployment Model toolset was designed as a unified and modular framework to set up the key components of such large-scale simulations. It does so by providing multiple implementations at each step, corresponding to various levels of fidelity. The toolset supports the generation of the simulated environment, graph-based optimizations for both static and dynamic deployments, the ingestion of various data sources for traffic dynamics, physics-based detector response functions and detection processes, as well as some performance estimation tools at both the sensor and network levels. As entirely physics-based computations at each simulation step are practically infeasible, the UDM toolset aims to provide performance estimates based on various levels of approximations while maintaining the integrity of the simulation.

## Data Availability

Road network and traffic data used for illustration is publicly available from OpenStreetMap and GoogleMaps servers.
